# Feeding Cues and Injected Nutrients Induce Acute Expression of Multiple Clock Genes in the Mouse Liver

**DOI:** 10.1371/journal.pone.0023709

**Published:** 2011-08-25

**Authors:** Hideaki Oike, Kanji Nagai, Tatsunobu Fukushima, Norio Ishida, Masuko Kobori

**Affiliations:** 1 National Food Research Institute, National Agriculture and Food Research Organization, Tsukuba, Japan; 2 Mitsubishi Rayon Company, Ltd., Yokohama, Japan; 3 Biomedical Research Institute, National Institute of Advanced Industrial Science and Technology, Tsukuba, Japan; 4 Graduate School of Life and Environmental Sciences, University of Tsukuba, Tsukuba, Japan; Vanderbilt University, United States of America

## Abstract

The circadian clock is closely associated with energy metabolism. The liver clock can rapidly adapt to a new feeding cycle within a few days, whereas the lung clock is gradually entrained over one week. However, the mechanism underlying tissue-specific clock resetting is not fully understood. To characterize the rapid response to feeding cues in the liver clock, we examined the effects of a single time-delayed feeding on circadian rhythms in the liver and lungs of *Per2::Luc* reporter knockin mice. After adapting to a night-time restricted feeding schedule, the mice were fed according to a 4, 8, or 13 h delayed schedule on the last day. The phase of the liver clock was delayed in all groups with delayed feeding, whereas the lung clock remained unaffected. We then examined the acute response of clock and metabolism-related genes in the liver using focused DNA-microarrays. *Clock* mutant mice were bred under constant light to attenuate the endogenous circadian rhythm, and gene expression profiles were determined during 24 h of fasting followed by 8 h of feeding. *Per2* and *Dec1* were significantly increased within 1 h of feeding. Real-time RT-PCR analysis revealed a similarly acute response in hepatic clock gene expression caused by feeding wild type mice after an overnight fast. In addition to *Per2* and *Dec1*, the expression of *Per1* increased, and that of *Rev-erbα* decreased in the liver within 1 h of feeding after fasting, whereas none of these clock genes were affected in the lung. Moreover, an intraperitoneal injection of glucose combined with amino acids, but not either alone, reproduced a similar hepatic response. Our findings show that multiple clock genes respond to nutritional cues within 1 h in the liver but not in the lung.

## Introduction

The mammalian circadian clock consists of a central pacemaker in the suprachiasmatic nucleus (SCN) of the hypothalamus and various oscillators in most peripheral tissues [1]. The molecular oscillator of the circadian clock is thought to depend on a negative transcriptional feedback loop of core clock genes such as *Per1*, *Per2*, *Cry1*, *Cry2*, *Clock* and *Bmal1* [2]. In addition to these genes, several other clock genes such as *Rev-erbα*, *Dbp*, *Dec1* and *Dec2*, reinforce the molecular oscillator of the transcriptional circuit [3], [4], [5]. The circadian clock is not only robust but it is also flexible enough to adapt to surrounding circumstances. Light-dark (LD) cycles comprise a critical cue for the central clock in the SCN, whereas cyclic feeding behavior is the predominant cue for many peripheral tissue clocks [6], [7]. Restricted daytime feeding obviously entrains the circadian clocks of many peripheral tissues in nocturnal animals and even in extra-SCN brain regions, even though SCN activity is locked to LD cues [8], [9], [10]. Although the phases of circadian gene expression are similarly shifted in most peripheral tissues after one week of daytime feeding, food-induced phase resetting proceeds faster in the liver than in the kidney, heart, pancreas or lung [8], [9]. Two days of restricted feeding efficiently shifts the phase of the liver clock in mice and rats [9], [11] and even a 30-min feeding stimulus induces rapid *Per2* and *Dec1* gene expression within 1 h in the rat liver and shifts the circadian phases of clock gene expression on the following day [12]. However, the molecular profile underlying the variable sensitivity of tissues to feeding cues and the nutrients required to affect the peripheral clocks remain obscure.

Findings from behavioral and cell culture experiments suggest that an increase in the glucose level is involved in feeding-induced entrainment [13], [14]. A 100% glucose diet causes food anticipatory activity (FAA) rhythms in mice and rats, whereas feeding on glucose alone does not entrain the mouse liver clock [11], [14]. In contrast, the oral intake of sugars plus proteins can entrain the liver clock in mice [11], indicating that a balanced diet is required for proper entrainment of this clock.

Enteral nutrition at restricted times entrains the circadian rhythm of blood cortisol in humans [15]. In contrast, total parenteral feeding despite a restricted duration abolishes the adrenocortical rhythm, although the blood urea level, which is signalled by the time of feeding, remains at the same level as that for oral feeding [16]. Moreover, jejunal resection attenuates the daily rhythm of corticosterone in rat blood [17]. These results suggest that nutritional digestion in the gastrointestinal region is critical to entrain peripheral clocks to feeding. However, this is controversial because total parenteral nutrition could entrain the clocks of the SCN and liver in rats [18].

Although feeding cues obviously entrain many peripheral clocks, studies of the molecular mechanism underlying the food entrainment of each peripheral clock are scant. The present study compares the response of the lung and liver clocks in mice to clarify the molecular profile underlying the rapid response of the liver clock to nutritional cues.

## Materials and Methods

### Animals and Handling

Animals were handled according to the guidelines of the Ministry of Agriculture, Forestry and Fisheries for laboratory animal studies and the study was reviewed and approved by the Animal Care and Use Committee of the National Food Research Institute, National Agriculture and Food Research Organization (NARO), Japan (approval ID; H21-083, 084, 092, H22-001 and 010).

BALB/cAn mice (males, 10–30 weeks) were obtained from the Institute for Animal Reproduction, Charles River Japan. Homozygous of the *Clock* mutant mice (Jcl:ICR genetic background) were described previously [19]. *Per2::Luc* knockin mice [20] established by Dr. Joseph Takahashi (Northwestern University, USA) were supplied by the Jackson Laboratory (USA) and bred as homozygotes. All mice were housed under 24±1°C, 55±5% humidity and a 12 h light-dark (LD) photocycle (light period from 08:00 to 20:00) with free access to water and a standard diet (NMF; Oriental Yeast, Japan).

### Luminescent Analysis of Explants from *Per2::Luc* Mice

Liver and lung explants were prepared [11], [21], [22] from male and female *Per2::Luc* mice (23–50 weeks). The circadian rhythmicity in the liver explants did not significantly differ between the sexes [23], [24]. The mice were sacrificed at ZT3 to record bioluminescence rhythmicity in the liver and the lung. The livers and the lungs were rapidly removed from the mice and placed in cold Hanks' balanced salt solution (HBSS; Sigma, USA) with 10 mM HEPES (Sigma) and penicillin-streptomycin (Sigma). The blocks were cut into pieces and explanted in 35-mm dishes, sealed with Parafilm and incubated at 37°C under a 5% CO_2_ atmosphere with DMEM containing high glucose (Invitrogen, USA), 10 mM HEPES, penicillin-streptomycin and 0.1 mM luciferin (Promega, USA). Bioluminescence was measured and integrated for 1 min at intervals of 10 min using a dish-type luminometer (Kronos Dio AB-2500; Atto, Japan).

Luminescence data were analyzed as described [11], [22], [25]. The original data were smoothed using an adjusting-averaging method with 100-min running means and then the dataset was detrended by subtracting the 24-h running average from the raw data. Peaks were defined as points at which the bioluminescence was higher than that on both sides of the points and confirmed from waveforms. The peak phase time was determined from the peak that initially appeared between 12 and 36 h of culture.

### DNA Microarray Analysis

We performed DNA microarray analysis using the fibrous DNA chip Genopal® (Mitsubishi Rayon, Japan) as described [22], [26], [27]. We synthesized DNA oligonucleotide probes to detect 206 genes related to metabolic processes and installed them in Genopal®, which comprises hollow plastic fibers containing a gel to which the probes can attach [28]. All probes (65-mer), including those for positive and negative controls, were designed to have an annealing temperature (Tm) ranging from 65°C to 75°C throughout the microarray.

We randomly selected 8 of 30 *Clock* mutant mice (males, 39–62 weeks) and placed them in cages equipped with infrared sensors (AS10D; Melquest, Japan) to record locomotor activity. All of the mice were bred under 12 h LD conditions for two weeks followed by constant light (LL) for three weeks. Behavioral data were visualized using CIF-II software (Melquest) and rhythmicity was evaluated using Metlab software (MathWorks Inc., USA). After breeding under LL for 3 weeks, the mice were sacrificed at 6, 12, 18, and 24 h after starting to fast and at 1, 2, 4 and 8 h after resumed feeding (that is, 25, 26, 28 and 32 h from the start of fasting) by cervical dislocation and then the livers and lungs were immediately collected in RNAlater™ (Ambion, USA). Total RNA was extracted from the excised tissues using the RNeasy Mini kit (Qiagen KK, Japan) and amplified using the MessageAmpII biotin-enhanced amplification kit (Applied Biosystems, USA) according to the manufacturer's instructions. Biotinylated aRNA was disintegrated using fragmentation reagents (Applied Biosystems) and then incubated at 95°C for 7.5 min. Hybridization, washing and fluorescent-labelling proceeded using the Genopal® UE-104 system (Mitsubishi Rayon). Hybridization was performed using a DNA microarray (Genopal®) in hybridization buffer, 0.12 M Tris-HCl/0.12 M NaCl/0.05% Tween-20, and the fragmented biotinylated aRNA at 65°C for 16 h. The hybridized DNA microarray was washed in 1 mL of 0.12 M Tris-HCl/0.12 M NaCl/0.05% Tween-20 at 65°C and in 1 mL of 0.12 M Tris-HCl/0.12 M NaCl. The microarray was then fluorescently labelled with streptavidin-Cy5 (GE Healthcare Bio-Sciences KK, Japan), washed in 0.12 M Tris-HCl/0.12 M NaCl/0.05% Tween-20 at room temperature and in 1 mL of 0.12 M Tris-HCl/0.12 M NaCl.

Hybridization signals were acquired using a DNA microarray reader and multibeam excitation technology (Yokogawa Electric Co., Japan). The DNA microarrays were scanned at multiple exposure durations of 0.1, 0.4, 1.0, 4.0, and 30 s. Intensity values with the optimal exposure condition for each spot were selected according to saturation. The relative amount of each transcript was normalized to the amount of 18S ribosomal RNA in the same sample.

### Quantitative RT-PCR

BALB/c mice fasted overnight from ZT9 resumed feeding from the next ZT 1, or were intraperitoneally injected with 0.3 mL of nutrients, 30% glucose (Glc; 1.7 M; 3.0–3.5 g/kg body weight), a mixture of 12 amino acids (12AA; 50× MEM amino acids solution (PromoCell GmbH, Germany) comprising L-isomers of 10 mM F, 10 mM H, 20 mM I, 20 mM K, 20 mM L, 5 mM M, 30 mM R, 20 mM T, 20 mM V, 2.5 mM W, 10 mM Y and 5 mM Cystine), Glc with 3 amino acids (3AA; L-isomers of F, M, and T; the concentrations of all of these were the same as in the mixture containing 12AA), Glc with 6 amino acids (6AA; L-isomers of K, H, I, L, R, and V; the concentrations of all of these were the same as in the mixture containing 12AA), Glc with 9 amino acids (9AA; mixture of 3AA and 6AA comprising L-isomers of F, K, H, I, M, L, R, T, and V) or PBS (control) at ZT1. The final osmolarity of each solution was not adjusted. The mice were then sacrificed by cervical dislocation at ZT2 for one-time sampling, or at ZT2, 5, 8, and 11 for multi-time sampling. The livers and lungs were immediately collected in RNAlater and total RNA was extracted described above. Reverse transcription proceeded using Superscript II reverse transcriptase (Invitrogen) with random hexamers according to the manufacturer's protocol. Gene-specific primers (Table 1) and Thunderbird SYBR qPCR Mix (Toyobo, Japan) were included in the real-time PCR protocol. The reaction proceeded at 95°C for 15 s, 60°C for 31 s and for 40 cycles. Amounts of PCR products were monitored using an ABI Prism 7000 sequence detection system and analyzed using ABI Prism 7000 SDS software (Applied Biosystems). All primer pairs spanned exon junctions. The relative amounts of each transcript were normalized to the amount of *GAPDH* transcript in the same cDNA.

**Table 1:** Primer pairs used for real-time RT-PCR.

Gene names	Forward (5′-3′)	Reverse (5′-3′)
***mPer1***	gcttcgtggacttgacacct	tgctttagatcggcagtggt
***mPer2***	caacacagacgacagcatca	tcctggtcctccttcaacac
***mDec1***	ccatgggttaggtgagccatg	tgtctgcacttagtagagtctgag
***mDec2***	ctacacacactctcagactgg	gtttctgtcctgtaatctgtgg
***mRev-erbα***	ccctggactccaataacaaca	tgccattggagctgtcact
***mDbp***	gcattccaggccatgagact	ccagtacttctcatccttctgt
***mGck***	gtgaggtcggcatgattgt	tccaccagctccacattct
***mGapdh***	catggccttccgtgttccta	cctgcttcaccaccttcttga

### Statistical Analysis

All values are expressed as means ± SEM. Differences in expression levels and peak times were statistically evaluated using Student's t-test for single comparisons, and one-way ANOVA with Dunnett's *post-hoc* test for multiple comparisons using Prism 5 software (GraphPad Software; USA).

## Results

### Single Feeding Delays Circadian Clock in the Liver but not in the Lung

**Figure 1:**
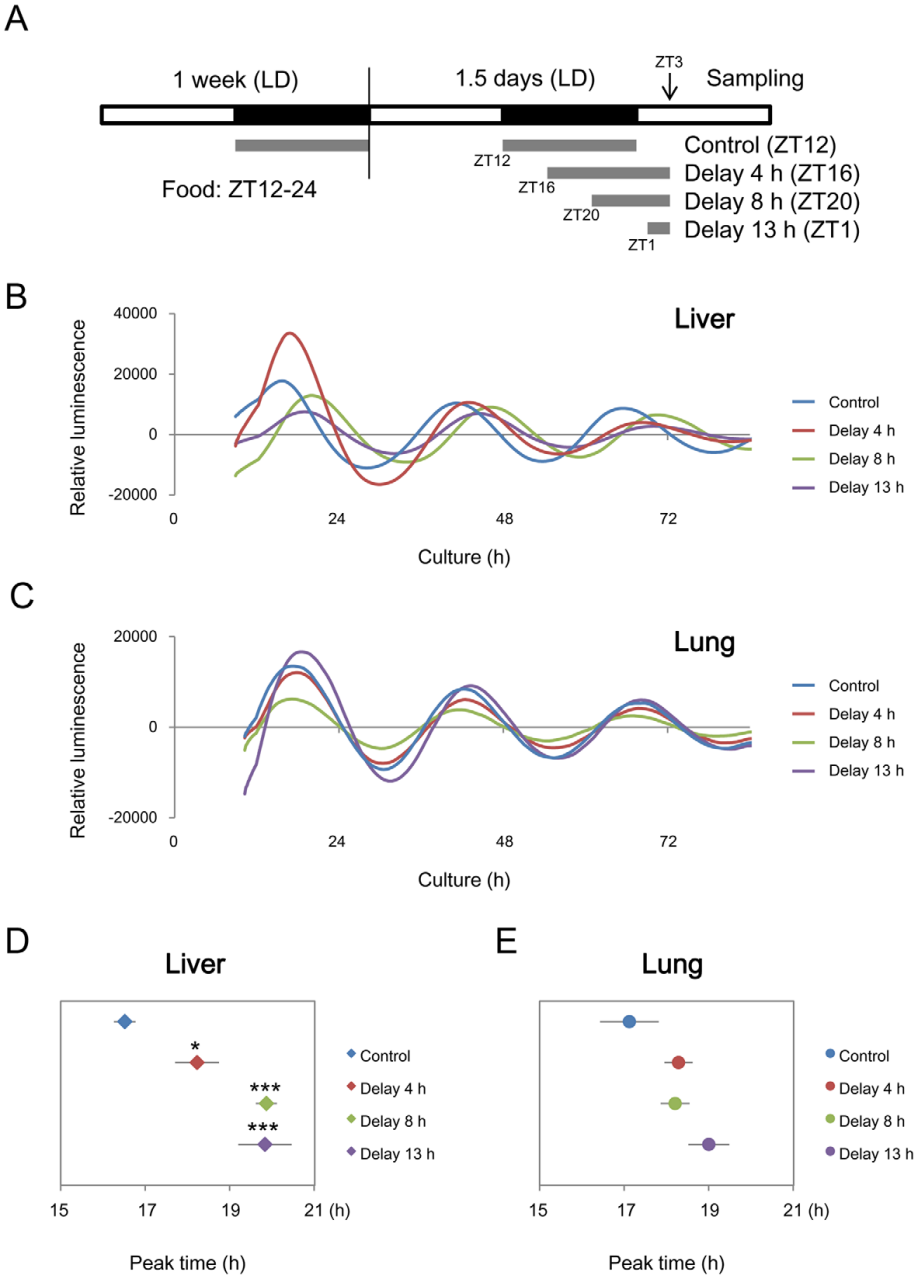
Single feeding delays phase of liver, but not lung clock. After a night-time restricted feeding schedule (ZT12-24) for one week, mice were divided into 4 groups and then delayed feeding was applied as follows. Mice in the 4, 8 and 13 h delayed groups were fed from ZT16, 20, and the next ZT1, respectively. Control mice were fed from ZT12 to ZT24. White and black boxes indicate day and night respectively; gray lines show feeding times (A). Representative circadian profiles of reporter luminescence in explants of livers (B) and lungs (C) isolated from *mPer2::Luc* knockin mice. Peak times of circadian oscillation between 12 and 36 h in culture are shown for liver (D) and lung (E). Values are shown as means ± SEM (n = 7 for ZT12 and ZT16, and n = 5 for ZT20 and ZT1; *p<0.05, ***p<0.001; vs. control; Dunnett's test).

To understand the response of peripheral clocks induced by feeding cues, we examined the effects of a single delayed feeding on the circadian clocks in the liver and lungs. *Per2::Luc* knock-in mice [20] received a night-time restricted feeding schedule (ZT12-24) for one week, and then the feeding start time was delayed on the last day by 0 (control), 4, 8 and 13 h; that is, feeding from ZT12, 16, 20 and on the next day at ZT1 (Figure 1A). Figure S1 shows representative results of temporal consumption suggesting that the mice consumed similar amounts or more food during feeding that was delayed by 4 h and 8 h to the controls, and that the mice with feeding delayed by 13 h consumed less food than the controls. The liver and lung explants were isolated from the mice at ZT3 and then circadian luminescence was recorded (Figure 1B and 1C). Peak time statistically differed in the liver among groups (one-way ANOVA, *p*<0.001, n = 5–7; Figure 1D). The peak times of the groups given delayed feeding differed from control values (*p*<0.05, Dunnett's test). In contrast, the peak time was not shifted in the lungs of all four groups (one-way ANOVA, *p*>0.05, n = 5–7; Figure 1E). These results indicated that a single feeding obviously delays the circadian clock in the liver, but not in the lungs.

### Acute Response of Clock Genes to Feeding in the Liver of *Clock* Mutant Mice

**Figure 2:**
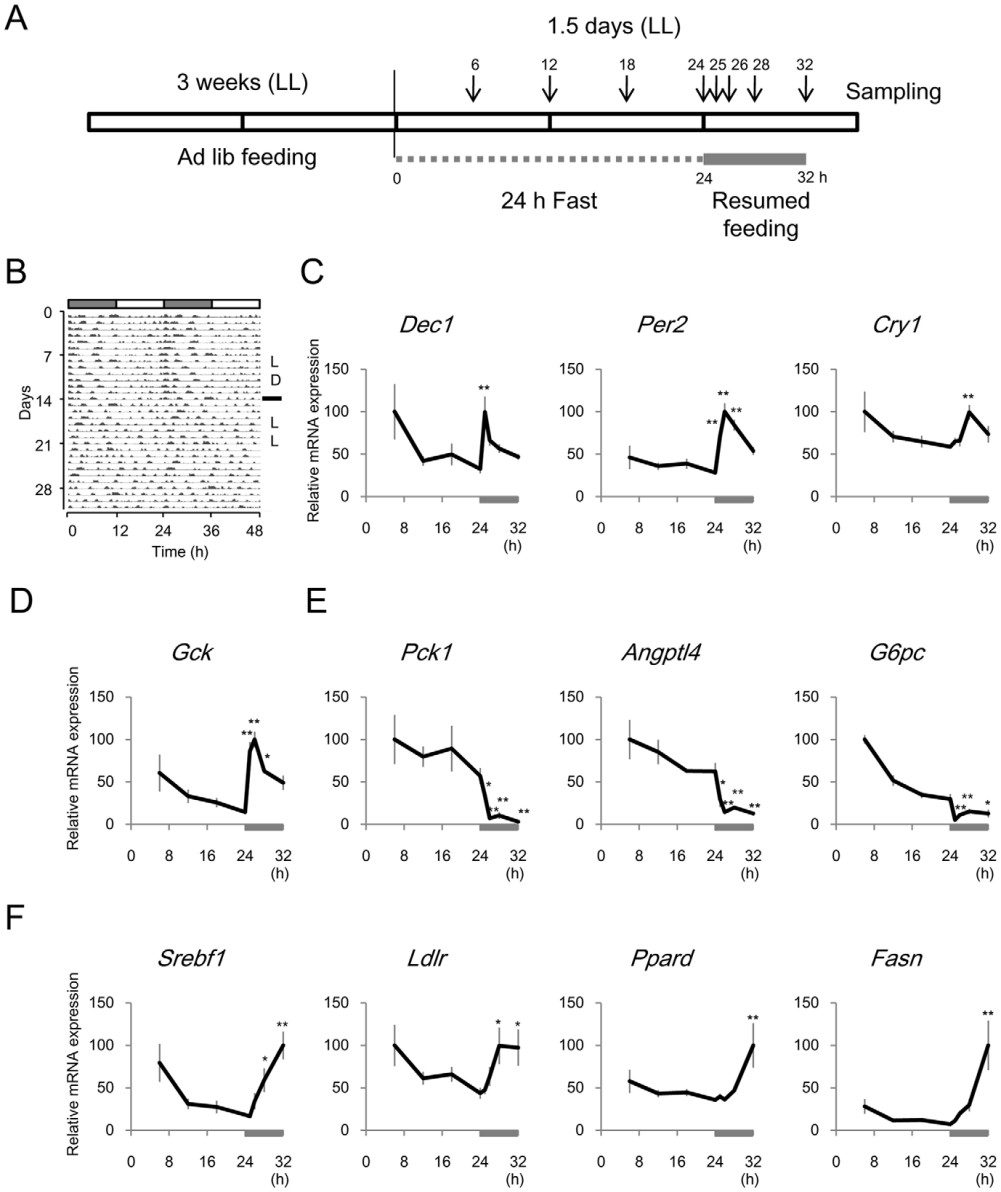
Rapid response of gene expression after resumed feeding in liver of *Clock* mutant mice. (A) Experimental design. *Clock* mutant mice were bred under constant light (LL) for 3 weeks, fasted for 24 h and then fed for 8 h. (B) Representative actogram from mice under 12-h light-dark (LD) cycle for 2 weeks that were switched to LL. (C–F) Expression profiles of mRNAs for clock genes (C), genes related to glucose metabolism (D–E), and fatty acid synthesis (F). Gray bars in both panels, duration of resumed feeding. Data are shown as means ± SEM (n = 5 for 25 h, n = 4 for 12, 18, 24 and 26 h, and n = 3 for 6, 28, and 32 h; *p<0.05, **p<0.01; 24 h vs. 25–32 h; Dunnett's test).

To characterize which genes are associated with rapid response of the liver clock induced by feeding, we performed DNA microarray analysis using a focused DNA chip containing 206 metabolic genes including 18 clock genes (Materials and Methods and Table S1). We minimized the endogenous circadian rhythm of gene expression in the liver by breeding *Clock* mutant mice under constant light (LL). In fact, the behavioural rhythms of the mice were attenuated after three weeks (Figure 2B). Moreover, *Clock* mutant mice are entrained normally by feeding cues [29], [30]. We examined the gene expression profile under 24 h of fasting and for 8 h after feeding was resumed (Figure 2A). The endogenous rhythm of clock gene expression was quite weak under 24 h fasting (Figure 2C and Table S1). Although the expression of most clock genes was not significantly affected within 8 h after feeding was resumed (one-way ANOVA, *p*>0.05), *Per2*, and *Dec1* were transiently induced within 1 h after resuming feeding and *Cry1* was slightly increased after 4 h (*p*<0.01; Figure 2C). With respect to other genes, *Gck* (glucokinase) was transiently induced within 1 h after resuming feeding as well as *Per2* and *Dec1* (*p*<0.01; Figure 2D). None of the genes on the DNA chip was acutely induced within 1 h after feeding like *Gck* (Table S1). Several genes related to glucose production such as *Pck1* (phosphoenolpyruvate carboxykinase 1), *Angptl4* (angiopoietin-like 4) and *G6pc* (glucose-6-phosphatase) were acutely decreased within 1 h (Figure 2E), whereas those associated with fatty acid synthesis such as *Ppard* (peroxisome proliferator activator receptor delta), *Srebf1* (sterol regulatory element binding transcription factor 1), *Ldlr* (low density lipoprotein receptor) and *Fasn* (fatty acid synthase) gradually increased for 8 h after feeding was resumed (Figure 2F).

### Acute Response of Clock Genes to Feeding in Wild Type Mice

**Figure 3:**
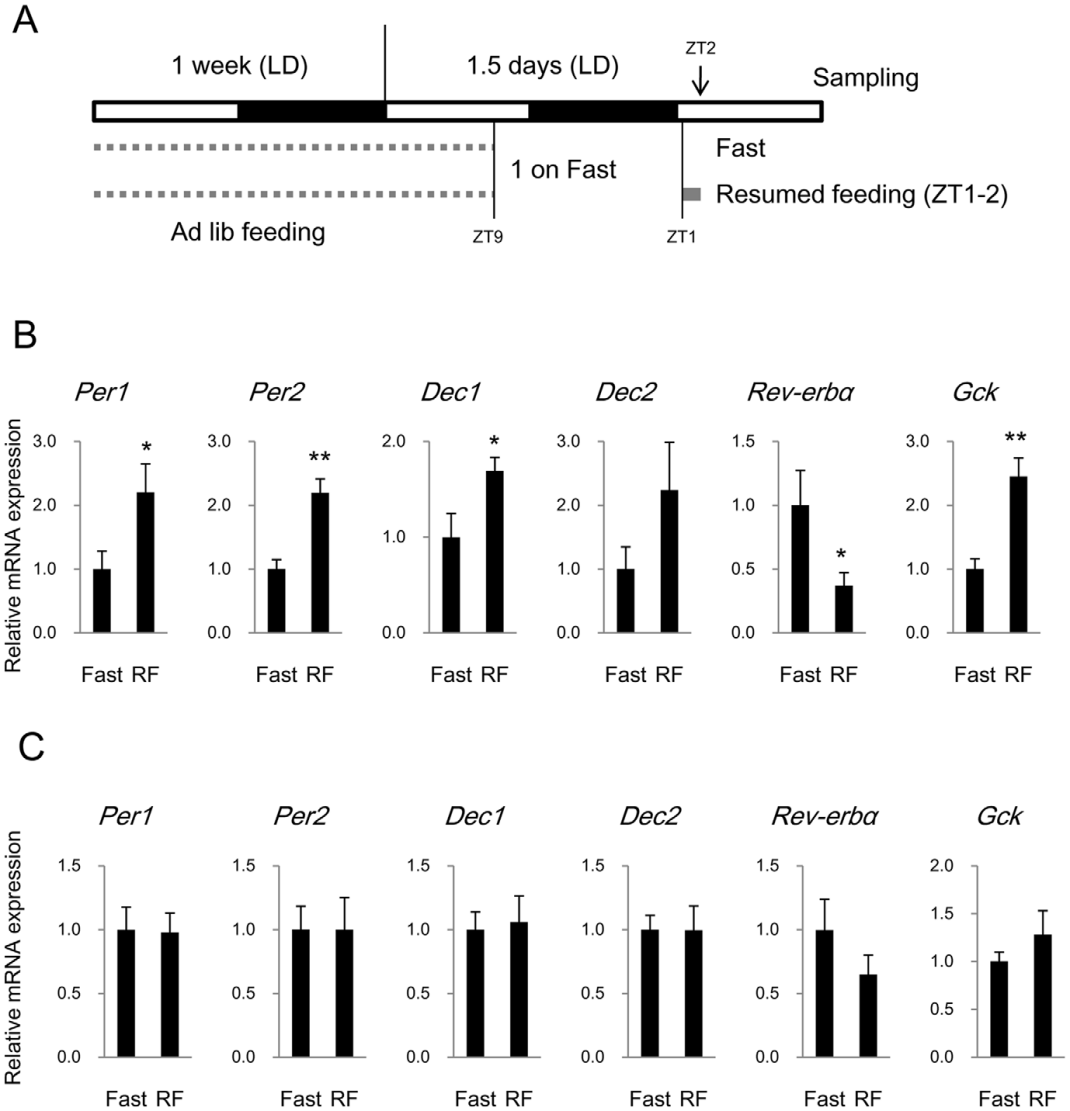
Rapid response in clock gene and *Gck* expression in liver and lung after resumed feeding. (A) Experimental design. BALB/c mice were fasted overnight and assigned to resumed feeding (RF). Mice in RF group resumed feeding for 1 h and those in Fast group (control) continued to fast. All animals were sampled at ZT2. (B and C) Expression of mRNAs for clock genes and *Gck* in liver (B) and lung (C) is shown as means ± SEM relative to control group (n = 8, *p<0.05, ** *p*<0.01; t-test).

We clarified the acute response of clock genes to feeding in wild type BALB/c mice using real-time RT-PCR analysis. The mice were fed at ZT1 after an overnight fast and then sampled at ZT2 (Figure 3A) because the microarray analysis showed that one hour is sufficient to induce the expression of early responsive genes such as *Gck*, *Dec1*, and *Per2* (Figure 2). Moreover, only 2 h feeding at ZT1 after an overnight fast delayed the liver clock (Figure 1), suggesting that the liver responds to feeding cues within 2 h. The expression of clock genes (*Per1*, *Per2*, *Dec1*, *Dec2*, and *Rev-erbα*) and *Gck* at ZT2 was examined in the liver and lungs. Levels of *Per1*, *Per2*, and *Dec1* rapidly increased within 1 h of feeding in the liver (*p*<0.05; Figure 3B), but not in the lung (Figure 3C). *Dec2* might also be increased in the liver of some mice, but a significant difference was undetected in this cohort. *Rev-erbα* was significantly decreased in the liver (*p*<0.05), but not in the lung, and *Gck* was increased in the liver (*p*<0.01), but not significantly altered in the lung. We also analyzed the expression of *Bmal1* and *Dbp* in the liver and found that it was not affected after 1 h of feeding (Figure S2). These results were consistent with the above findings using *Clock* mutant mice.

### Intraperitoneal Injection of Nutrients Induces Acute Resetting of Clock in Liver

**Figure 4:**
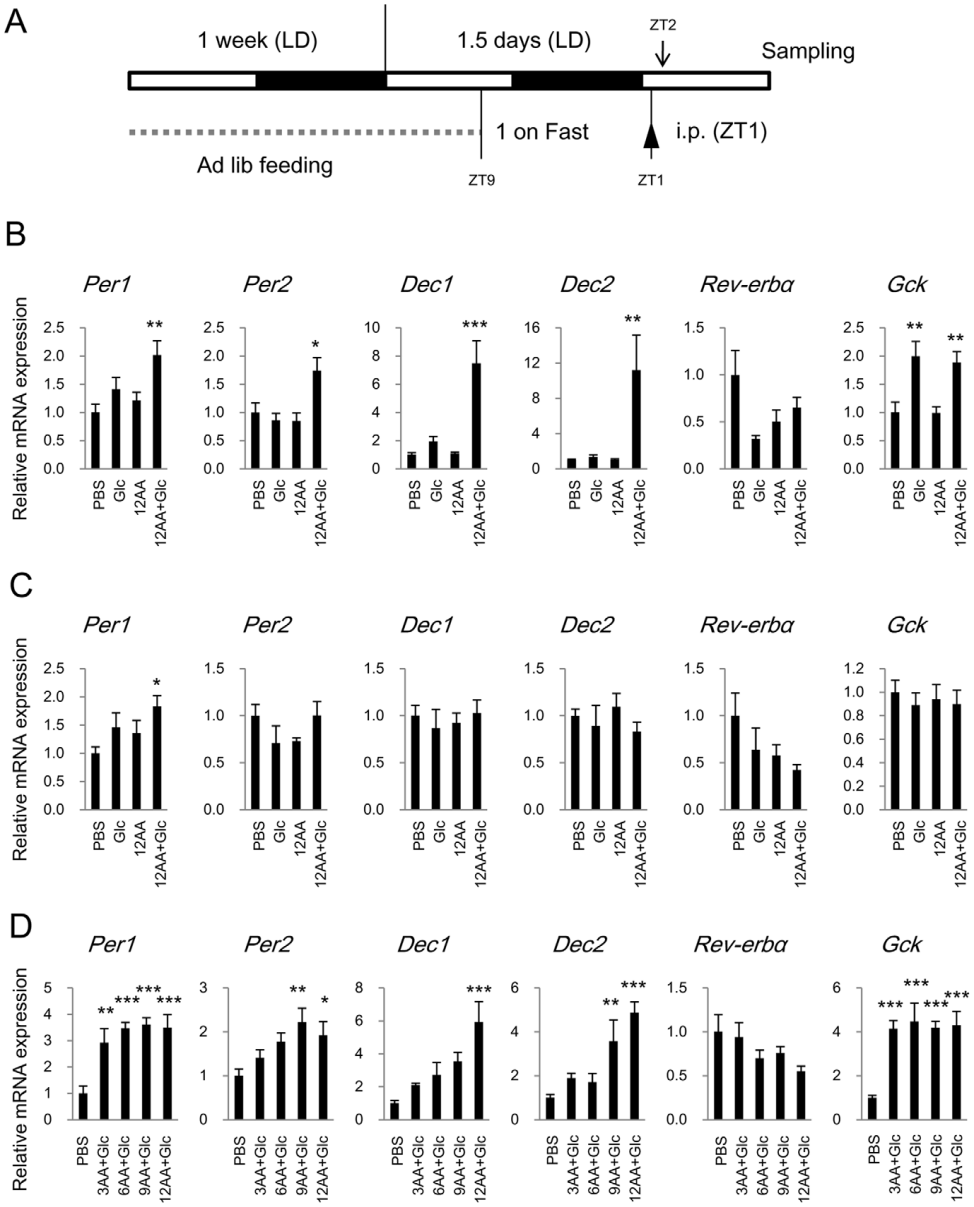
Rapid response in clock gene and *Gck* expression in liver and lung after intraperitoneal injection of nutrients. (A) Experimental design. BALB/c mice were fasted overnight then intraperitoneally injected with nutrients (Glc: glucose, AA, amino acids; see Materials and methods) or PBS (control) at ZT1. All animals were sampled at ZT2. (B–D) Expression of clock gene and *Gck* mRNA in liver (B and D) and lung (C) is shown as means ± SEM relative to control group (n = 8, *p<0.05, **p<0.01, ***p<0.001; vs. control; Dunnett's test).

Short term feeding within 1 h induced a rapid response of clock genes in the mouse liver. We examined whether an intraperitoneal injection of nutrients also induces such a response in the liver to determine whether or not intestinal digestion is necessary and which nutrient is required for the rapid response. We used the same time course that was applied in the previous experiment. That is, mice were injected at ZT1 after an overnight fast with 30% glucose (Glc), a mixture of 12 amino acids including all essential amino acids (12AA; L-isomers of F, H, I, K, L, M, R, T, V, W, Y and Cystine), or a mixture of both (12AA+Glc) and then the liver and lungs were sampled at ZT2 (Figure 4A). We found that 12AA+Glc induced *Per1*, *Per2*, *Dec1* and *Dec2* expression in the liver within 1 h of injection (Figure 4B) as well as resumed feeding after an overnight fast. In contrast, an injection of either Glc or 12AA did not affect hepatic expression of these clock genes. Glc and Glc+12AA induced *Gck* in the liver but not in the lung (Figure 4B and C). Glc+12AA slightly induced *Per1* in the lung, but did not affect *Per2*, *Dec1*, and *Dec2* (Figure 4C). Levels of *Rev-erbα* tended to decrease in the liver and lung after an injection of any of the nutrients, but the difference did not reach statistical significance.

We characterized the amino acid requirement in more detail using three more mixtures generated by subtracting amino acids from the 12AA mixture: 3AA (F, M and T), 6AA (H, I, K, L, R, and V), and 9AA (mixtures 3 plus 6). The time course experiment was repeated in another cohort of BALB/c mice (Figure 4A) that was fasted overnight and then divided into groups that were injected with PBS (control) or Glc+3AA, Glc+6AA, Glc+9AA or Glc+12AA at ZT1. All nutrient mixtures induced *Per1* and *Gck* expression (*p*<0.01 vs. PBS; Figure 4D). Both Glc+9AA and Glc+12AA, but not Glc+3AA and Glc+6AA induced *Per2*, and *Dec2*, and only Glc+12AA induced *Dec1* mRNA. Although none of the injected mixtures statistically decreased *Rev-erbα*, the average expression level gradually declined dependently upon the numbers of injected AA. These results showed that an injection of glucose combined with amino acids elicited an acute response of clock genes in the liver but not in the lung.

Finally, to examine that this acute response induced by an injection of nutrients actually causes a phase delay of the liver clock, we determined the temporal expression of *Rev-erbα* and *Dbp* in liver and lung on the next daytime after the nutrients injection (Figure S3A). We used these two clock genes because they sharply peak in the daytime [8], [27], [31], [32], which facilitates detection of the phase shift. Moreover, *Dbp* would be an appropriate indicator to show the phase of liver clock because it was not acutely affected by the nutritional injection but its expression was expected to be affected in the following cycle as a clock controlled gene. The injection of 12AA+Glc significantly affected *Rev-erbα* expression around the peak time of the following day in the liver but not in the lung (Figure S3B and C). The expression increased at ZT2 and 5, and decreased at ZT8. Tendency was similar for *Dbp* (increased at ZT5 and 8, and decreased at ZT11), but without reaching statistical significance. These results seem that an intraperitoneal injection of 12AA+Glc caused phase delay of the liver clock in the following day.

## Discussion

Our results showed that a single feeding and a single intraperitoneal injection of nutrients can efficiently induce an acute response of clock genes in the liver but not in the lung, and that a combination of glucose and amino acids is required for this response.

The mice seemed to consume a similar amount or more food during feeding that was delayed by 4 and 8 h to controls, and only the mice with feeding delayed by 13 h seemed to consume less food than the others because the time until sampling was shorter. Despite the decreased food consumption, our results indicated that only 2 h of feeding (feeding delayed by 13 h) is sufficient to delay the mouse liver clock. Moreover, a single intraperitoneal injection of nutrients containing fewer calories than the total daily food intake elicited a response from liver clock genes. Consuming less than the total daily amount of food might entrain the liver clock at least under fasting conditions. This is consistent with a recent study showing that only 30 min of feeding stimuli are sufficient to significantly induce the expression of *Per2* and *Dec1* within 1 h and alter the transcript levels and circadian phases of other clock genes including *Rev-erbα* in the liver of rats after a longer interval [12]. The phase delay was larger in the 8- and 13-h, than in the 4-h group. This seemed to fit with phase-response curves as well as the response of the central clock to light [33]. Delayed feeding for 13 h is close to the turnaround point because daytime restricted feeding from ZT5 (17 h-delayed) induces a phase advance of the peripheral clock in the mouse [34]. Consequently, the maximum delay caused by a single feeding might be around 4 h.

The expression rhythms of clock- and metabolism-related genes in the liver were attenuated in *Clock* mutant mice bred under constant light, whereas the circadian expression of about 50 of the 200 genes on this DNA chip was detectable in wild type mice under 12 h LD [27]. This is a useful way to evaluate responses to various stimuli, as it can distinguish them from intrinsic circadian changes in expression. In fact, the microarray data showed that feeding cues inhibit genes related to gluconeogenesis (*Pck1*, *Angptl4*, and *G6pc*), gradually increase the genes associated with fatty acid synthesis (*Srebf1*, *Ldlr*, *Ppard*, and *Fasn*), and induce acute expression of the clock genes, *Per2* and *Dec1*, among 200 genes related to metabolism in the liver. Insulin secretion caused by glucose uptake in the blood acutely increases *Gck* and decreases *Pck1*, genes that are associated with glucose metabolism [35], [36], [37]. Indeed, we found that the feeding cue caused rapid *Gck* induction and a rapid *Pck1* decrease in the liver of *Clock* mutant mice. Moreover, *Gck* was also induced by a glucose injection in the livers of wild type mice, which was consistent with the fact that an intraperitoneal injection of glucose induces insulin secretion [38]. However, the expression of clock genes including *Per2* and *Dec1* was not affected by the glucose injection, suggesting that another pathway induces these clock genes. Such unknown signalling, which is probably modulated by protein modifications such as phosphorylation and acetylation because of the rapidity, might include key molecules that link nutritional signals to circadian rhythms in the liver.

We found here that an intraperitoneal injection of glucose together with amino acids induced rapid changes in clock gene expression in the liver but not in the lungs. We could not exclude the possibility that total osmolarity affects rapid responses to stimuli in the liver. However, the osmolarity of the glucose solution was much higher than that of the amino acid mixture, and it did not induce a rapid response of clock gene expression, suggesting that the response depends on nutritional components rather than on osmolarity. Our results are consistent with the findings that a diet of either glucose or protein alone does not influence the phase of liver clock whereas a diet containing both sugar and protein delays the phase of liver clock [11].

The 12 amino acids that we used are components of Minimal Essential Medium (MEM), that includes all of the essential amino acids and in which mammalian cells are generally cultured. It is possible to record circadian luminescent rhythms from cultured liver samples obtained from *Per2*::*Luc* mice for several days using MEM. In fact, injecting 12 amino acids with glucose induces acute clock gene expressions and also affected expression of *Rev-erbα* even after 24 to 36 h in the liver, suggesting that these amino acids comprise a sufficient nutritional cue for the liver. Moreover, we analyzed the contribution of each of the 12 amino acids classified into 3 groups based on properties such as having a basic or branched chain (HKRILV), hydrophobicity (YW and Cystine), and other (FMT). The combination of 3, 6, 9 and 12 amino acids with glucose induced *Per1* expression, and that of 9 and 12 amino acids with glucose also stimulated *Per2* and *Dec2* expression. The combination of 3 and 6 amino acids with glucose induced expression to some degree although the values did not reach statistical significance. Similarly, all combinations might induce *Dec1* and suppress *Rev-erbα*, but without reaching statistical significance. This implies that any amino acid included in the experiment together with glucose might be sufficient to induce clock gene expression.

Our observations provide an important insight into the rapid response of the liver clock to feeding cues. Namely, digestion or nutrient sensing in the intestine is unnecessary, whereas glucose plus amino acids are essential. Our results suggest that glucose and amino acids digested from proteins circulating in the blood are critical nutrient cues for liver clock. Rapid resetting of the liver clock induced by feeding is associated with the acute induction of *Per2* and *Dec1* transcription in rats [12]. Our results are consistent with *Per2* and *Dec1* induction and we also found changes in *Per1*, *Dec2*, and *Rev-erbα* in the liver after short-term feeding and after injecting nutrients. Multiple clock genes are coincidentally induced during rapid resetting of the mammalian liver clock, indicating that this clock is closely associated with energy metabolism. In fact, a deficiency in liver clock function causes low blood glucose levels in fasting phase [39]. On the other hand, nutritional signals are not as critical for the lungs to induce the acute response because the lung clock was not affected by delayed feeding, short-term resumed feeding, and injected nutrients except for *Per1* induction by Glc+12AA. Body temperature also works as a universal resetting cue for peripheral clocks [40], and thus the lung clock might be gradually reset by a body temperature rhythm that depends on feeding schedules.

Peripheral clocks dominate not only local physiology but also energy metabolism and hormonal secretion at the whole body level [39], [41]. Because nutritional cues are critical for peripheral clock entrainment, biological rhythms disrupted by irregular feeding might lead to various physical and mental disorders [1], [7]. Understanding the mechanisms underlying the entrainment of peripheral clocks with food is linked to the prevention of many lifestyle-related diseases and to improving the quality of life.

## Supporting Information

Figure S1 **Representative data of temporal food consumption immediately before sampling in experiment shown** **Figure 1** **.** Bars show amount of food consumed during 15 min. Y axis shows 0.5 g. Total onsumption is shown at right of graph. Another independent assay showed total consumption of 2.3 g (Control), 2.7 g (Delayed 4 h), 2.5 g (Delayed 8 h), and 1.5 g (Delayed 13 h).
(TIF)

Figure S2 ***Dbp*** **and** ***Bmal1*** **expression in liver 1 h after resumed feeding.** Expression of mRNA for *Dbp* in liver under the condition in shown Figure 3. Means ± SEM (n = 8). There was no significant difference in both genes.
(TIF)

Figure S3 **Intraperitoneal injection of nutrients delays phase of liver, but not lung clock.** (A) Experimental design. BALB/c mice fasted overnight and then were intraperitoneally injected with nutrients (12AA+Glc) or PBS (control) at ZT1. All animals were sampled at next day ZT2, 5, 8, and 11. (B and C) Temporal expression of *Rev-erbα* and *Dbp* mRNA in liver (B) and lung (C) is shown as means ± SEM (n = 3, *p<0.05, **p<0.01 Student's t-test).
(TIF)

Table S1 **Gene expression data in DNA microarray analysis.**
(PDF)
