# Supplementary figures and images for: Feeding Cues and Injected Nutrients Induce Acute Expression of Multiple Clock Genes in the Mouse Liver
Source: PLoS One. 2011 Aug 25;6(8):e23709. doi: 10.1371/journal.pone.0023709 (PMC3162004; doi:10.1371/journal.pone.0023709)

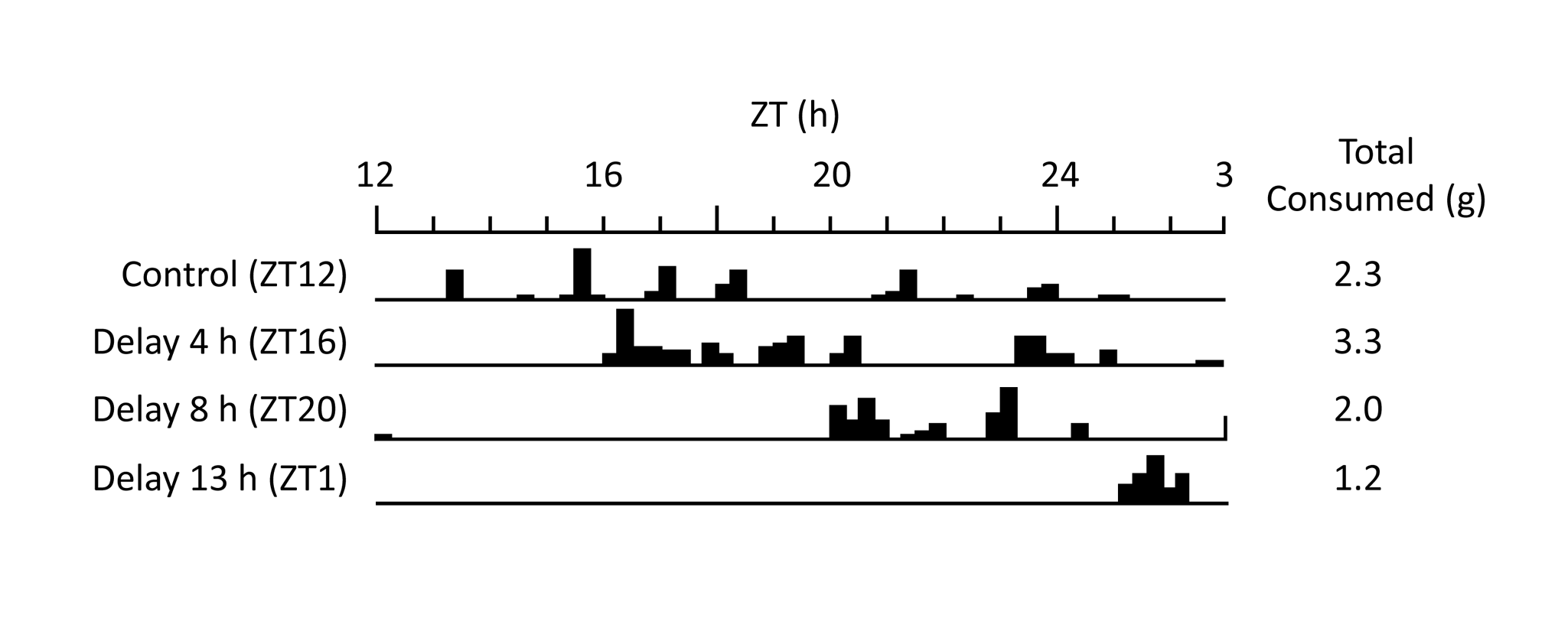

Supplement: Figure S1 — Representative data of temporal food consumption immediately before sampling in experiment shown Figure 1 . Bars show amount of food consumed during 15 min. Y axis shows 0.5 g. Total onsumption is shown at right of graph. Another independent assay showed total consumption of 2.3 g (Control), 2.7 g (Delayed 4 h), 2.5 g (Delayed 8 h), and 1.5 g (Delayed 13 h). (TIF) [file pone.0023709.s001.tif]

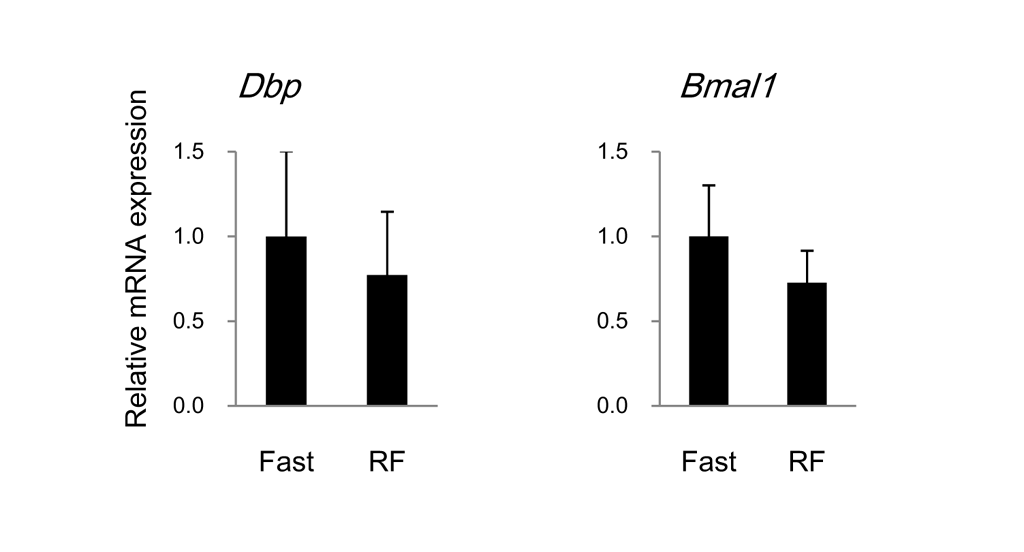

Supplement: Figure S2 — Dbp and Bmal1 expression in liver 1 h after resumed feeding. Expression of mRNA for Dbp in liver under the condition in shown Figure 3. Means ± SEM (n = 8). There was no significant difference in both genes. (TIF) [file pone.0023709.s002.tif]

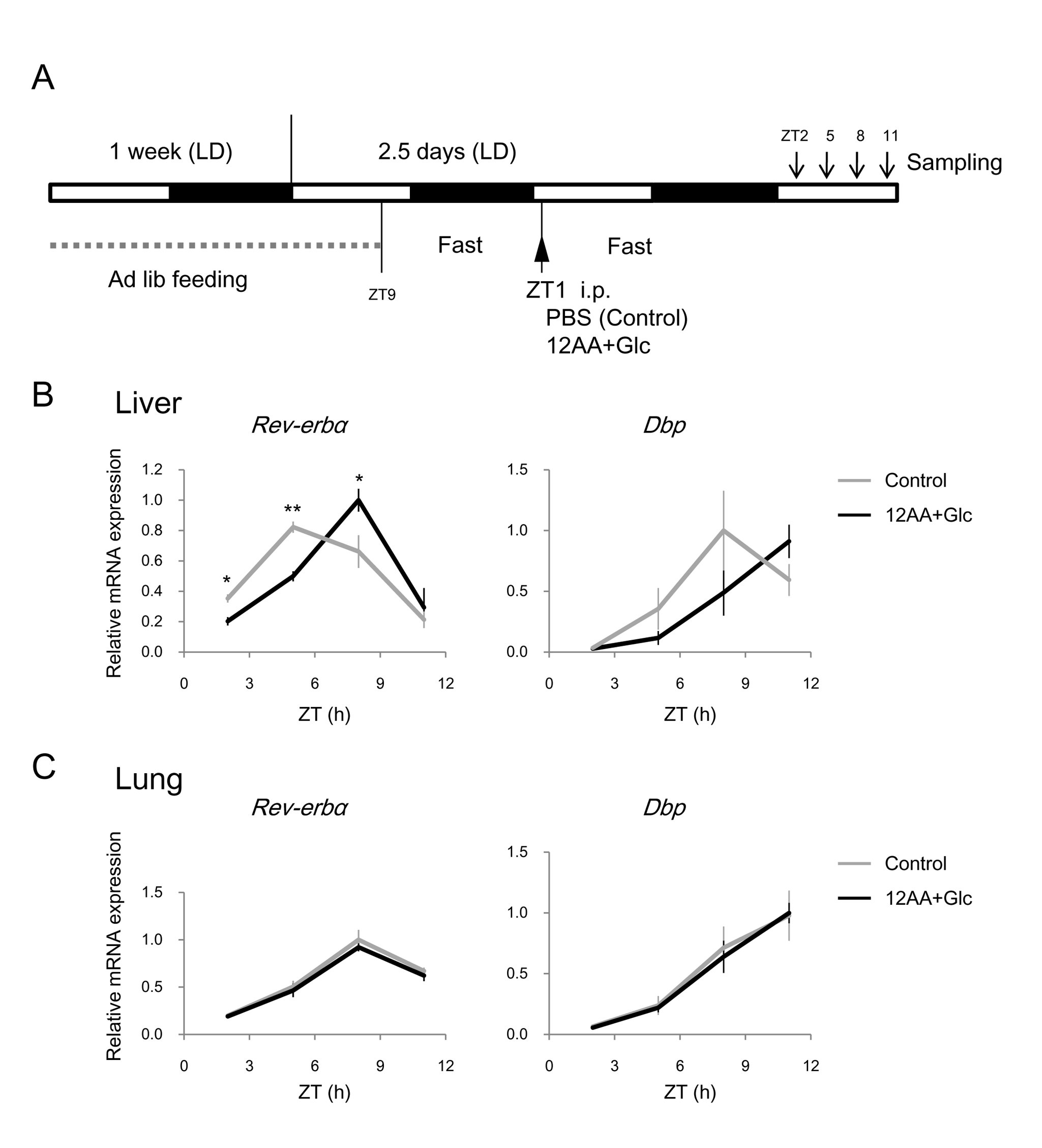

Supplement: Figure S3 — Intraperitoneal injection of nutrients delays phase of liver, but not lung clock. (A) Experimental design. BALB/c mice fasted overnight and then were intraperitoneally injected with nutrients (12AA+Glc) or PBS (control) at ZT1. All animals were sampled at next day ZT2, 5, 8, and 11. (B and C) Temporal expression of Rev-erbα and Dbp mRNA in liver (B) and lung (C) is shown as means ± SEM (n = 3, *p<0.05, **p<0.01 Student's t-test). (TIF) [file pone.0023709.s003.tif]
