# Supplementary material for: Feeding Cues and Injected Nutrients Induce Acute Expression of Multiple Clock Genes in the Mouse Liver
Source: PLoS One. 2011 Aug 25;6(8):e23709. doi: 10.1371/journal.pone.0023709 (PMC3162004; doi:10.1371/journal.pone.0023709)
Supplement: Table S1 — Gene expression data in DNA microarray analysis. (PDF) [file pone.0023709.s004.pdf]

		Average (Max = 100)									SE									One way ANOVA (24h-32h)		
Accession	Gene Symbol	6 h	12 h	18 h	24 h	25 h	26 h	28 h	32 h		6 h	12 h	18 h	24 h	25 h	26 h	28 h	32 h		*p*	**p*<0.05<br>***p*<0.01	Dunnett's test<br>*p*<0.05 (vs 24 h)
NM_011076	Abcb1a	97	65	100	60	63	56	66	84		25	7	48	11	5	2	10	28		0.171		
NM_008830	Abcb4	100	82	84	62	93	72	69	40		50	12	17	12	17	9	16	6		0.243		
NM_133360	Acaca	100	50	46	30	29	37	49	59		28	6	11	1	1	4	4	8		0.000	**	32 h
NM_007382	Acadm	100	69	78	64	67	64	82	46		36	6	9	10	4	3	15	7		0.443		
NM_007383	Acads	100	59	58	42	48	42	52	37		31	6	5	5	3	3	5	5		0.173		
NM_207624	Ace	100	68	62	47	60	56	65	47		27	5	4	2	6	2	5	6		0.314		
NM_015729	Acox1	100	47	58	42	47	37	45	24		32	7	12	2	5	7	11	8		0.226		
NM_053115	Acox2	100	67	70	61	74	58	69	54		24	7	6	6	10	4	6	8		0.154		
NM_030721	Acox3	100	64	61	48	58	53	62	40		31	5	4	1	7	2	5	5		0.210		
NM_007981	Acsl1	100	81	66	51	63	60	66	27		41	13	8	13	8	4	5	8		0.325		
NM_001033600	Acsl4	83	50	62	47	85	74	100	90		15	4	19	10	33	7	9	13		0.191		
NM_080575	Acss1	100	79	67	62	71	70	71	53		26	5	8	4	6	6	2	4		0.384		
NM_007393	Actb	78	60	68	57	60	87	98	100		11	8	19	11	6	15	4	11		0.008	**	32 h
NM_009605	Adipoq	100	72	62	59	60	65	67	48		29	6	7	3	4	3	2	4		0.189		
NM_197985	Adipor2	100	63	56	39	46	48	56	46		13	7	5	2	5	3	6	5		0.126		
NM_007428	Agt	100	79	77	64	61	58	65	46		24	5	6	8	2	5	1	5		0.272		
NM_009647	Ak3l1	100	69	57	48	48	54	55	45		32	6	14	5	4	1	7	3		0.220		
NM_013913	Angptl3	100	57	52	54	38	48	51	44		23	6	5	3	4	4	4	4		0.062		
NM_020581	Angptl4	100	85	63	62	30	14	20	13		23	14	2	10	9	1	1	2		0.000	**	25, 26, 28, 32 h
NM_020581	Angptl4	100	95	71	61	35	18	28	15		29	15	1	13	10	1	2	2		0.001	**	25, 26, 28, 32 h
NM_009703	Araf	100	82	85	65	66	62	78	59		26	3	16	5	10	3	3	3		0.225		
NM_007475	Arbp	100	82	79	74	83	84	100	85		15	3	8	3	3	2	9	3		0.085		
NM_007489	Arntl (Bmal1)	99	67	72	68	73	66	100	78		20	8	8	7	10	5	13	12		0.064		
NM_172309	Arntl2	100	76	67	56	75	73	82	53		21	10	5	4	7	4	8	6		0.385		
NM_016774	Atp5b	100	66	67	51	54	53	61	52		27	6	9	5	8	2	4	3		0.047	*	
NM_020615	Atp5c1	100	63	65	47	47	51	62	51		33	4	7	4	4	1	6	4		0.020	*	
NM_025983	Atp5e	100	79	70	58	66	61	63	61		10	4	7	1	3	1	4	5		0.088		
NM_016755	Atp5j	100	69	62	56	59	57	55	53		14	3	4	2	3	3	2	5		0.082		
NM_138597	Atp5o	100	69	61	51	54	56	61	56		19	5	2	3	4	2	1	4		0.047	*	
NM_018794	Atp6ap1	100	66	81	64	66	71	74	59		31	6	21	2	7	4	4	5		0.139		
NM_016920	Atp6v0a1	100	61	58	51	58	53	70	66		21	6	2	3	5	2	2	11		0.020	*	
NM_033617	Atp6v0b	100	55	67	44	54	47	57	40		33	5	18	6	6	4	5	2		0.445		
NM_013477	Atp6v0d1	100	61	66	48	51	55	68	50		36	4	13	3	6	3	4	6		0.148		
NM_007509	Atp6v1b2	100	74	85	68	65	58	67	55		27	4	24	8	9	2	4	6		0.226		
NM_007510	Atp6v1e1	100	72	82	62	63	61	68	55		27	5	21	3	10	3	2	1		0.125		
NM_025381	Atp6v1f	100	77	72	64	66	69	70	69		13	2	5	2	3	2	3	3		0.049	*	
NM_133826	Atp6v1h	100	68	75	55	59	69	87	55		33	7	17	4	6	5	8	5		0.188		
NM_011498	Bhlhb2 (Dec1)	100	42	50	33	100	66	57	47		33	6	13	5	18	6	5	3		0.007	**	25 h
NM_024469	Bhlhb3 (Dec2)	21	16	16	12	100	20	19	11		5	2	2	1	56	2	4	1		0.182		
NM_139294	Braf	100	71	71	58	78	79	77	52		31	5	6	3	6	6	5	5		0.307		
NM_011333	Ccl2/MCP1	88	71	92	100	67	75	69	84		24	6	34	47	5	14	4	31		0.553		
NM_007678	Cebpa	100	79	68	51	65	63	68	47		23	5	5	4	5	5	4	8		0.446		
NM_021455	ChREBP/Mlxipl	100	50	49	35	31	31	35	34		31	3	8	1	4	3	3	5		0.022	*	26 h
NM_007715	Clock	100	75	68	56	74	71	77	53		25	7	6	3	9	5	8	4		0.277		
NM_007743	Col1a2	98	77	89	100	63	61	67	69		22	6	18	37	4	6	4	20		0.376		
NM_009931	Col4a1	100	64	83	79	62	58	76	78		19	5	19	24	6	2	2	20		0.311		
NM_007744	Comt	100	68	60	40	41	64	65	39		21	7	2	3	3	7	9	5		0.797		
NM_001017429	Cox17	100	83	76	70	75	82	85	76		11	4	4	4	6	2	6	5		0.102		
NM_009941	Cox4i1	100	72	68	61	65	64	61	55		16	3	2	4	3	2	1	4		0.130		
NM_007747	Cox5a	100	71	63	55	66	65	63	63		17	4	3	1	8	2	1	2		0.068		

NM_009942	Cox5b	100	77	67	58	72	66	73	68		11	6	6	2	4	4	3	4		0.058		
NM_007748	Cox6a1	100	80	76	61	69	68	71	60		9	5	7	3	4	4	7	5		0.202		
NM_009943	Cox6a2	100	79	80	60	69	68	77	53		19	6	6	2	4	4	2	5		0.202		
NM_007751	Cox8b	100	93	68	58	65	67	77	53		28	19	3	3	6	7	3	9		0.635		
NM_013495	Cpt1a	96	83	100	84	86	54	44	22		29	8	9	5	20	4	4	2		0.003	**	26, 28, 32 h
NM_009948	Cpt1b	100	80	72	57	63	66	79	73		26	10	6	3	6	3	21	21		0.168		
NM_009949	Cpt2	100	80	75	61	81	79	95	67		23	11	3	7	4	5	6	11		0.388		
NM_007771	Cry1	100	71	65	59	65	66	99	73		24	7	7	2	4	7	9	10		0.003	**	32 h
NM_009963	Cry2	100	72	74	62	53	52	60	39		28	7	3	5	6	4	4	5		0.024	*	26 h
NM_026444	Cs	100	69	60	52	57	71	89	68		33	9	7	4	7	6	10	4		0.051		
NM_009993	Cyp1a2	100	63	38	40	32	50	41	26		33	5	4	4	5	6	6	7		0.600		
NM_024264	Cyp27a1	100	72	62	51	45	54	48	39		30	7	5	4	5	3	3	6		0.043	*	28 h
NM_021282	Cyp2e1	100	84	74	67	66	73	70	30		35	7	16	11	4	6	5	7		0.084		
NM_017396	Cyp3a41a	100	44	45	37	22	36	30	28		44	4	15	4	2	4	4	6		0.153		
NM_010011	Cyp4a10	39	32	36	49	83	55	100	39		23	7	4	11	20	3	29	5		0.136		
NM_177406	Cyp4a12a	100	54	42	29	32	39	40	31		31	6	6	2	3	3	4	10		0.869		
NM_007824	Cyp7a1	27	45	36	64	47	100	93	42		11	9	17	23	11	7	29	11		0.678		
NM_016974	Dbp	100	74	72	55	66	58	67	45		27	5	3	3	6	2	6	5		0.096		
NM_026172	Decr1/Nadph	100	66	64	50	64	57	72	45		30	7	5	6	7	5	12	9		0.423		
NM_007922	Elk1	100	71	66	55	64	60	68	50		27	6	5	1	5	3	2	5		0.185		
NM_017399	Fabp1	100	76	56	36	42	48	52	41		29	6	9	12	6	4	10	13		0.815		
NM_007988	Fasn	28	12	12	7	11	20	30	100		9	1	3	1	2	4	7	29		0.000	**	32 h
NM_019395	Fbp1	100	46	58	43	51	50	52	34		41	3	5	4	4	5	8	7		0.538		
NM_010197	Fgf1	100	84	73	62	60	63	85	49		38	11	6	12	2	5	9	12		0.399		
NM_020013	Fgf21	14	18	28	20	53	100	16	10		3	5	3	5	36	31	2	2		0.180		
NM_010234	Fos	100	72	78	63	85	65	78	54		26	6	12	4	15	3	6	4		0.178		
NM_008061	G6pc	100	52	35	30	5	11	15	13		5	6	4	6	1	2	3	5		0.001	**	25, 26, 28, 32 h
NM_019468	G6pd2	100	40	72	31	28	35	30	44		49	16	52	10	15	8	24	25		0.520		
NM_008084	Gapdh	84	57	59	49	56	56	72	100		18	4	8	5	4	6	7	5		0.001	**	32 h
NM_010292	Gck	61	33	26	14	86	100	63	49		22	8	5	2	11	9	1	8		0.004	**	25, 26, 32 h
NM_013528	Gfpt1	72	72	58	47	65	81	100	56		13	20	19	13	13	3	36	4		0.678		
NM_013529	Gfpt2	100	74	45	52	60	65	51	49		34	42	22	8	9	9	28	6		0.482		
NM_010294	Gk2	91	100	37	35	51	65	44	26		23	41	14	3	9	13	10	14		0.661		
NM_010271	Gpd1	100	83	56	41	59	63	84	68		15	9	5	7	6	10	3	15		0.119		
NM_010274	Gpd2	100	60	39	27	44	58	48	45		45	7	8	3	3	8	6	11		0.185		
NM_008163	Grb2	100	80	77	64	58	58	64	60		20	5	10	6	2	2	7	2		0.068		
NM_007952	Grp58	48	23	27	24	37	59	100	66		17	1	10	3	5	7	13	9		0.000	**	28, 32 h
NM_008182	Gsta2	100	54	45	36	11	30	25	74		21	9	23	18	2	4	4	55		0.316		
NM_010357	Gsta4	100	57	46	38	23	36	41	53		19	5	11	6	2	1	3	13		0.047	*	
NM_008194	Gyk	100	80	70	61	73	71	79	45		27	10	5	6	5	2	8	4		0.294		
NM_030678	Gys1	100	68	46	43	69	74	56	53		26	17	16	8	21	4	4	6		0.701		
NM_145572	Gys2	100	65	55	50	70	48	55	33		43	2	1	7	26	3	7	5		0.458		
NM_173371	H6pd	100	70	72	51	58	37	37	25		31	4	13	4	17	3	3	3		0.056		
NM_178878	Hadha	100	76	71	53	63	59	70	50		27	7	4	7	4	5	7	8		0.387		
NM_010438	Hk1	100	72	68	53	74	73	65	52		28	9	19	2	14	7	3	4		0.700		
NM_008255	Hmgcr	60	44	44	30	34	35	65	100		14	3	9	3	3	1	27	63		0.159		
NM_145942	Hmgcs1	30	17	17	10	11	10	33	100		9	4	3	3	3	2	23	82		0.198		
NM_009327	HNF1	79	68	76	60	90	98	100	69		16	3	5	6	9	10	10	8		0.445		
NM_008284	Hras1	100	74	63	52	57	62	74	68		17	3	6	3	1	5	1	2		0.044	*	
NM_008289	Hsd11b2	100	70	60	49	58	55	63	45		28	5	3	1	5	1	3	5		0.210		
NM_010548	Il10	100	66	57	48	57	56	64	41		28	6	3	2	5	4	5	5		0.649		
NM_031168	Il6	100	65	56	46	63	63	74	44		27	7	6	2	5	4	10	5		0.525		
NM_010591	Jun	89	84	88	64	100	97	78	72		27	12	21	5	11	8	4	14		0.347		

NM_010699	Ldha	100	65	98	68	84	71	84	58		35	5	22	14	11	5	22	13		0.387		
NM_008492	Ldhb	100	62	72	57	68	58	70	49		37	5	10	10	7	2	13	9		0.244		
NM_013580	Ldhc	100	57	49	29	61	59	21	34		38	6	15	11	8	9	10	6		0.140		
NM_010700	Ldlr	100	61	66	44	48	63	100	97		24	7	9	6	5	11	22	21		0.003	**	32 h
NM_008493	Lep	100	69	60	47	60	59	69	45		28	5	4	1	5	3	6	4		0.326		
NM_008280	Lipc	100	69	50	47	40	44	46	30		27	4	10	6	3	3	3	3		0.227		
NM_008509	Lpl	82	100	66	68	43	41	36	66		23	15	29	23	10	4	4	37		0.387		
NM_008928	Map2k3	100	65	64	50	66	67	91	66		24	7	4	2	4	3	5	6		0.052		
NM_011840	Map2k5	100	79	73	61	57	52	51	43		26	7	8	3	5	2	5	3		0.049	*	26, 28 h
NM_011944	Map2k7	100	78	69	57	72	70	81	58		28	7	2	3	8	5	7	5		0.222		
NM_001081292	Map3k10	100	71	65	54	70	67	77	50		25	6	3	1	5	3	3	5		0.430		
NM_016896	Map3k14	100	76	75	59	75	70	70	49		22	8	9	3	5	2	4	4		0.252		
NM_011946	Map3k2	100	72	67	55	65	63	70	49		27	5	3	1	5	3	5	5		0.282		
NM_011947	Map3k3	100	78	75	62	68	66	71	52		25	5	9	5	6	4	2	2		0.375		
NM_016693	Map3k6	100	68	69	54	81	58	73	43		36	6	4	3	18	8	5	3		0.174		
NM_011950	Mapk13	100	82	75	64	78	76	81	56		27	8	5	4	7	5	9	4		0.248		
NM_172632	Mapk4	100	71	70	52	63	67	68	40		28	8	8	4	5	3	4	5		0.680		
NM_015806	Mapk6	100	74	71	60	58	51	66	57		24	8	10	3	5	2	3	2		0.029	*	26 h
NM_008551	Mapkapk2	100	80	79	66	80	74	73	58		18	7	17	7	15	8	2	6		0.555		
NM_008618	Mdh1	100	52	43	38	35	39	49	46		30	4	7	2	6	6	7	4		0.004	**	
NM_008617	Mdh2	100	58	54	50	66	58	80	74		31	2	11	8	10	7	11	7		0.006	**	32 h
NM_001033713	Mef2a	100	74	71	54	62	59	64	49		27	6	6	1	6	2	5	3		0.113		
NM_021462	Mknk2	99	74	100	60	64	37	42	33		30	3	35	5	12	3	2	8		0.058		
NM_010876	Ncf1	100	76	71	57	65	63	76	67		24	5	11	5	6	2	14	13		0.161		
NM_010877	Ncf2	100	66	82	66	60	57	76	92		25	2	21	10	6	1	20	19		0.026	*	
NM_010885	Ndufa2	100	81	75	69	76	77	79	72		5	4	7	2	3	4	6	4		0.185		
NM_025348	Ndufa3	100	84	74	65	72	70	71	63		11	5	11	2	3	2	6	5		0.210		
NM_010886	Ndufa4	100	74	73	60	54	60	62	55		11	4	4	3	1	1	3	5		0.063		
NM_025987	Ndufa6	100	82	75	74	76	73	72	64		10	5	4	6	7	3	4	6		0.091		
NM_026610	Ndufb4	100	79	66	58	63	62	70	66		8	6	7	2	3	1	5	4		0.039	*	
NM_145518	Ndufs1	100	55	58	45	51	48	68	50		36	5	7	3	8	4	10	5		0.040	*	
NM_153064	Ndufs2	100	64	60	45	47	45	53	45		25	4	4	3	5	2	3	5		0.023	*	
NM_133666	Ndufv1	100	60	57	41	48	45	52	43		38	5	7	5	5	2	4	6		0.115		
NM_028388	Ndufv2	100	66	61	54	58	57	65	60		22	4	3	3	7	3	2	6		0.013	*	
NM_017373	Nfil3	100	59	62	56	30	40	47	33		41	7	7	8	3	7	8	5		0.005	**	25, 26, 28 h
NM_010907	Nfkbia	100	81	91	81	40	43	45	49		24	5	22	11	7	7	1	9		0.030	*	25, 26, 28 h
NM_172203	Nox1	100	67	58	45	52	50	57	43		28	8	4	3	4	5	5	4		0.234		
NM_015760	Nox4	100	75	58	58	49	53	50	27		38	6	10	9	2	5	9	6		0.006	**	26, 28, 32 h
NM_008719	Npas2	100	67	60	50	58	61	66	43		31	6	5	3	5	4	5	9		0.593		
NM_011850	Nr0b2	100	58	68	29	11	17	39	36		42	7	24	9	3	2	5	4		0.018	*	
NM_145434	Nr1d1 (Rev-erba)	100	73	71	52	64	65	63	46		28	6	6	3	5	4	3	4		0.188		
NM_011584	Nr1d2	100	63	61	52	84	28	25	17		39	6	6	9	33	1	3	2		0.049	*	
NM_013839	Nr1h3	100	63	68	53	64	61	84	67		25	3	11	4	7	3	3	8		0.082		
NM_009108	Nr1h4	100	72	65	56	46	64	70	47		34	8	3	5	4	2	4	9		0.255		
NM_009803	Nr1i3	100	85	65	69	54	67	65	39		39	6	13	12	7	6	5	5		0.387		
NM_011044	Pck1	100	80	89	57	34	7	10	3		29	12	27	9	5	2	4	0		0.000	**	25, 26, 28, 32 h
NM_028994	Pck2	100	36	21	25	24	19	10	27		47	10	14	6	10	4	4	9		0.220		
NM_008810	Pdha1	100	49	46	44	42	45	56	46		41	5	6	6	6	2	13	5		0.064		
NM_013743	Pdk4	100	87	90	56	49	45	55	53		30	18	28	8	4	1	7	3		0.055		
NM_011065	Per1	86	76	79	72	100	64	94	36		17	8	14	13	27	4	11	3		0.166		
NM_011066	Per2	46	36	39	28	71	100	84	54		14	4	6	2	9	10	6	5		0.038	*	25, 26, 32 h
NM_011067	Per3	100	89	76	66	78	76	81	54		37	10	4	9	9	2	7	6		0.173		
NM_008824	Pfkfb1	100	57	67	54	83	54	72	51		31	6	11	3	22	4	11	9		0.205		

NM_133232	Pfkfb3	100	73	66	53	72	66	75	51		25	6	4	0	6	1	5	4		0.191		
NM_008826	Pfkl	100	67	64	51	56	57	64	53		24	5	10	3	3	2	3	4		0.119		
NM_001081274	Pgd	100	62	83	53	58	60	74	90		23	4	28	5	5	3	11	4		0.001	**	32 h
NM_013631	Pklr	100	66	56	48	58	69	74	64		32	6	5	3	3	6	7	11		0.151		
NM_011099	Pkm2	61	65	100	70	44	47	37	81		9	7	64	27	5	11	4	39		0.297		
NM_008898	POR	69	64	74	58	100	60	55	43		23	5	8	10	32	4	5	4		0.149		
NM_011144	Ppara	100	64	62	38	43	36	36	29		35	7	3	3	3	1	3	6		0.058		
NM_011145	Ppard	58	43	45	36	40	36	47	100		14	4	4	2	3	2	3	26		0.000	**	32 h
NM_011146	Pparg	100	72	64	53	52	60	66	50		26	14	8	4	4	8	9	3		0.452		
NM_008904	Ppargc1a	100	73	78	62	75	63	65	50		29	5	14	6	8	3	7	6		0.145		
NM_010936	PXR/Nr1i2	100	81	69	68	65	60	56	56		14	6	8	5	8	3	7	5		0.048	*	
NM_001099624	Rapgef2	100	69	83	63	58	61	71	52		32	5	24	7	8	5	6	3		0.266		
NM_009024	RARa	100	68	61	47	57	53	63	47		28	5	5	1	5	3	5	5		0.124		
NM_011243	RARb	100	70	62	52	64	60	70	56		29	6	6	2	6	3	8	4		0.058		
NM_013646	Rora	100	56	59	46	64	46	52	41		44	5	10	7	12	3	6	8		0.081		
NM_001043354	Rorb	100	82	76	62	73	76	76	54		23	8	6	8	4	6	5	2		0.605		
NM_011281	Rorc	100	66	71	59	67	76	76	50		32	6	9	4	5	4	3	4		0.740		
NM_011299	Rps6ka2	100	73	69	61	67	64	71	51		26	6	4	2	5	2	5	6		0.113		
NM_011305	RXRa	100	76	72	59	62	55	60	44		19	3	7	3	6	1	3	3		0.076		
NM_011306	RXRb	100	76	76	55	66	59	61	44		22	6	10	2	10	3	3	4		0.154		
NM_009127	Scd1	100	34	29	12	6	12	11	20		31	9	11	2	2	2	3	3		0.007	**	
NM_023281	Sdha	100	60	56	42	43	44	49	36		35	6	7	5	6	4	3	3		0.171		
NM_025321	Sdhc	100	58	53	42	45	45	57	49		30	3	7	3	3	1	2	4		0.055		
NM_008871	Serpine1	79	64	100	64	83	74	70	61		26	9	47	12	9	10	2	6		0.563		
NM_019812	Sirt1	100	68	68	56	58	50	58	39		28	6	11	3	6	3	8	5		0.055		
NM_022432	Sirt2	100	58	78	60	57	54	62	49		21	4	14	3	3	6	1	9		0.051		
NM_007451	Slc25a5	100	65	71	53	54	60	74	67		34	5	16	4	5	1	7	5		0.008	**	32 h
NM_011400	Slc2a1	100	77	82	54	65	60	84	57		30	7	13	1	5	7	7	4		0.121		
NM_031197	Slc2a2	100	55	31	32	26	33	46	50		36	5	8	8	5	4	11	4		0.007	**	32 h
NM_011401	Slc2a3	100	70	48	47	50	53	60	52		31	5	4	5	3	3	7	5		0.084		
NM_009204	Slc2a4	100	63	56	47	58	57	61	46		34	5	4	2	7	3	3	4		0.300		
NM_008063	Slc37a4	70	68	63	51	100	74	91	52		26	11	6	11	42	2	11	7		0.642		
NM_009270	Sqle	56	40	48	28	33	33	63	100		17	4	12	3	5	4	30	71		0.231		
NM_011480	Srebf1	80	31	28	18	16	34	59	100		22	6	7	1	2	9	14	16		0.000	**	32 h
NM_017376	Tef	100	94	82	69	58	55	53	30		41	11	8	15	4	3	5	5		0.011	*	26, 28, 32 h
NM_011577	Tgfb1	100	72	71	59	60	60	69	55		26	3	10	7	5	4	3	1		0.354		
NM_011593	Timp1	100	68	73	56	61	62	71	49		27	5	9	3	5	3	4	5		0.293		
NM_013693	Tnf	100	67	71	53	59	60	71	49		29	6	15	6	6	6	7	2		0.677		
NM_009422	Traf2	100	71	65	49	60	57	71	52		28	4	11	3	5	3	4	3		0.210		
NM_009463	Ucp1	100	68	60	51	66	65	70	46		34	5	3	3	5	3	3	4		0.471		
NM_011671	Ucp2	100	71	63	51	60	58	68	46		27	5	3	1	5	3	5	6		0.286		
NM_009464	Ucp3	100	69	78	62	69	70	75	54		29	6	10	1	5	7	4	3		0.217		
NM_025650	Uqcr	100	81	74	61	68	70	70	66		10	4	8	1	3	3	6	4		0.183		
NM_026219	Uqcrb	100	75	62	57	60	63	64	63		9	5	4	3	4	2	3	2		0.054		
NM_025407	Uqcrc1	100	63	57	41	53	47	57	45		37	6	6	7	6	4	4	6		0.200		
NM_025899	Uqcrc2	100	55	56	45	47	45	57	45		39	5	8	5	5	1	6	5		0.048	*	
NM_025710	Uqcrfs1	100	60	62	49	53	53	62	51		22	4	5	2	5	2	4	5		0.065		
NM_016808	Usp2	100	82	66	59	74	61	79	46		22	9	3	3	3	3	6	4		0.142		
NM_001025250	Vegfa	100	57	61	52	69	62	60	51		27	4	7	4	5	3	3	3		0.050		
